# Fast and label-free intraoperative discrimination of malignant pancreatic tissue by attenuated total reflection infrared spectroscopy

**DOI:** 10.1117/1.JBO.28.4.045004

**Published:** 2023-04-28

**Authors:** Rimante Bandzeviciute, Gerald Steiner, Katja Liedel, Jonas Golde, Edmund Koch, Thilo Welsch, Christoph Kahlert, Daniel E. Stange, Marius Distler, Jürgen Weitz, Justinas Ceponkus, Valdas Sablinskas, Christian Teske

**Affiliations:** a Institute of Chemical Physics, Faculty of Physics, Vilnius University, Vilnius, Lithuania; b Department of Visceral, Thoracic and Vascular Surgery, Faculty of Medicine and University Hospital Carl Gustav Carus, Technische Universität Dresden, Dresden, Germany; c Department of Anaesthesiology and Critical Care Medicine, Clinical Sensoring and Monitoring, Faculty of Medicine, Technische Universität Dresden, Dresden, Germany; d National Center for Tumor Diseases (NCT/UCC), Dresden, Germany; e German Cancer Research Center (DKFZ), Heidelberg, Germany; f Faculty of Medicine and University Hospital Carl Gustav Carus, Technische Universität Dresden, Dresden, Germany; g Helmholtz-Zentrum Dresden-Rossendorf (HZDR), Dresden, Germany; h Department of General, Visceral und Thoracic Surgery, St. Elisabethen-Klinikum Ravensburg, Academic Teaching Hospital of the University of Ulm, Ravensburg, Germany

**Keywords:** fiber-based spectroscopy, pancreas, attenuated total reflection infrared, infrared spectroscopy, pancreatic tumor

## Abstract

**Significance:**

Pancreatic surgery is a highly demanding and routinely applied procedure for the treatment of several pancreatic lesions. The outcome of patients with malignant entities crucially depends on the margin resection status of the tumor. Frozen section analysis for intraoperative evaluation of tissue is still time consuming and laborious.

**Aim:**

We describe the application of fiber-based attenuated total reflection infrared (ATR IR) spectroscopy for label-free discrimination of normal pancreatic, tumorous, and pancreatitis tissue. A pilot study for the intraoperative application was performed.

**Approach:**

The method was applied for unprocessed freshly resected tissue samples of 58 patients, and a classification model for differentiating between the distinct tissue classes was established.

**Results:**

The developed three-class classification model for tissue spectra allows for the delineation of tumors from normal and pancreatitis tissues using a probability score for class assignment. Subsequently, the method was translated into intraoperative application. Fiber optic ATR IR spectra were obtained from freshly resected pancreatic tissue directly in the operating room.

**Conclusion:**

Our study shows the possibility of applying fiber-based ATR IR spectroscopy in combination with a supervised classification model for rapid pancreatic tissue identification with a high potential for transfer into intraoperative surgical diagnostics.

## 1 Introduction

Pancreatic surgery is highly demanding and mainly performed to treat benign and malignant lesions of the pancreas. Due to the complexity of these procedures, the patient’s postoperative morbidity remains an important factor for the performance. Especially in pancreatic surgery for malignant lesions, a radical resection of the tumor in combination with sparing of physiological tissue is the main focus to maintain the organ’s endo- and exocrine function and therefore the patient’s quality of life. However, pancreatic ductal adenocarcinoma (PDAC) remains one of the most lethal entities even in localized diseases, with an average overall survival of 28 to 54 months.^1^^–^^3^ In advanced stage PDAC, lymph node and margin resection statuses are both leading factors for patient outcome.^4^

Tumor resection is often a balancing act between removing suspicious tissue and preserving the functional relevant tissue. In the marginal area of the tumor, the infiltrating cellular border is macroscopically invisible, often accompanied by inflammatory tissue.^5^ Thus, it is highly demanding for the surgeon to macroscopically differentiate among solid tumors, infiltrated area, and normal pancreatic tissues during surgery. Simultaneous pathologic evaluation of a frozen margin section is currently the state of the art to ensure tumor-free resection borders. However, it is rather unsuitable for complete tumor delineation due to a consecutive prolongation of the surgical intervention. Therefore, numerous approaches, such as magnetic resonance imaging, ultrasound, and 5-aminolevulinic acid for fluorescence-guided surgery, are used intraoperatively mainly in a study fashion to improve delineation of several tumor entities and to optimize the extent of resection.^6^ However, the precise identification of PDAC tumor borders and infiltrating areas in the retroperitoneal margin and the porto-mesenteric axis remains challenging. Those aids did not find their way into clinical routine. Hence, other objective techniques based on molecular characteristics need to be integrated into surgical procedures to achieve a rapid stain- and section-free margin evaluation and diagnosis *in situ*.

During the past decade, several studies have been published demonstrating methods of vibrational spectroscopy, such as infrared (IR) and Raman spectroscopy, with the potential to provide relevant diagnostic information on pancreatic tumors.^7^^,^^8^ They are well suited for clinical use due to being pure optical, damage-free techniques not requiring tissue processing or staining. Moreover, concepts for intraoperative spectroscopic analysis have been developed to provide information about the molecular composition of tissue that may be exploited for resection guidance.^9^ Furthermore, IR spectroscopy was shown to have the potential to detect recurrent solid cancer even before histopathological analysis displayed abnormalities in the tissue.^10^ Most of these studies only included a small number of resected and dried tissue samples in analytical laboratories far away from concrete clinical application. In one of the first papers on fiber optic IR spectroscopy of tumor tissue, Ollesch et al.^11^ demonstrated that spectroscopic differentiation between normal and tumorous colon tissue is possible on native *ex situ* samples using supervised classification. The recently published trend in analytical chemistry report also describes the need and trend for intraoperative tumor delineation using optical spectroscopic techniques.^12^ Nevertheless, the potential of vibrational spectroscopy has already been demonstrated for *in vivo* applications in neurosurgery and confirmed as a real-time diagnostic method.^13^^–^^15^ Lately, IR spectroscopy was proven to be suitable for the differentiation of PDAC, pancreatitis, and normal pancreatic tissue in frozen tissue sections.^16^ Hence, the next step toward an intraoperative application in pancreatic surgery is the analysis of freshly resected tissue using IR fiber optics.

Here, we report the results of the first study of native, unprocessed tissue samples of 58 patients to delineate tumor tissue from pancreatitis and normal pancreatic tissue during surgery. Attenuated total reflection (ATR) IR absorption spectra captured from small amounts of freshly resected pancreatic tissue indicate the presence of tumor, pancreatitis, or normal tissue in less than 1 min. Three out of the 58 patients were included in a successful intraoperative case study for tissue class differentiation during surgery. Therefore, we posit an approach for clinical and intraoperative spectra analysis for a comprehensive benchmarking framework of significant clinical scope to improve surgical technique.

## 2 Materials and Methods

### 2.1 Sample Preparation

Pancreatic tissue samples were collected from pancreatic surgical resections at the Department of Visceral, Thoracic and Vascular Surgery of the University Hospital Carl Gustav Carus, Technische Universität Dresden. Surgical procedures were performed for medically indicated reasons, and no additional tissue was resected for research interests. Freshly resected specimens were examined by a board-certified pathologist. Representative parts with macroscopically unequivocal features of “normal,” “tumor,” or “pancreatitis” tissue were extracted on ice, and corresponding tissue spectra were consecutively measured by ATR IR fiber spectroscopy. Standard histology reports of the whole resection specimens were reviewed for all samples, and classification diagnosis was set accordingly. Tumor tissues consisted of PDAC and several other (pre-) malignant entities infiltrating or located in the pancreas. In total, tissue samples of 58 patients (43 males and 15 females with a median age of 67.5, reaching from 19 to 84 years) were analyzed (Table S1 in the Supplementary Material).

### 2.2 ATR IR Fiber Spectroscopy

Measurements of freshly resected human pancreatic tissue spectra were performed. The measuring system consisted of an ATR silver halide fiber probe (Art Photonics GmbH, Berlin, Germany) attached to an FT-IR spectrometer Alpha (Bruker Optik GmbH, Ettlingen, Germany), equipped with an external liquid nitrogen cooled mercury–cadmium–telluride detector (Model IRA-20-00131, Infrared Associates, Inc.). The beam path in the Ge ATR crystal was calculated using the program “Optics Software for Layout and Optimization,” version 6.44 (Lambda Research, Littleton, Massachusetts, United States). The simulation of the ray paths was performed with 30,000 rays. Positions and angles of rays emerging from the spectrometer optical fiber were chosen randomly in each case within the allowable ranges. The IR fibers have a diameter of 850±20  μm and a numerical aperture of 0.3±0.03. As refractive index for the Ge crystal n=4 was set. Details of the system are described elsewhere.^17^^,^^18^

The cone-shaped Ge ATR crystal tip was pressed onto the sample tissue during the measurement covering a surface of $∼5\;{\mathrm{mm}}^{2}$. Before each measurement, tip cleaning was performed by wiping the tip surface by a soft cotton swab dampened with distilled water followed by ethanol. Cleanliness of the tip was clarified by measuring the spectrum of ambient air and comparing it to the background spectrum measured before the experiment. For each sample, several tissue slices were cut, and spectra were measured from these freshly cut surfaces. The measurements were labeled as individual spectra in this paper.

Fifty interferograms were recorded at a resolution of $4\;{\mathrm{cm}}^{-1}$, averaged and Fourier transformed to single beam spectrum using Blackmann-Haris 3-term apodization function, zero filling factor of 2, and calculation of power spectra from the interferograms.

### 2.3 Optical Coherence Tomography Imaging

For optical coherence tomography (OCT) imaging of the ATR crystal, a commercial spectrometer-based polarization-sensitive OCT system (TEL220PSC2, Thorlabs GmbH, Germany) with a broad spectral range of ∼1300  nm and a telecentric scanning lens (LSM03, Thorlabs GmbH, Germany) was used. After 3D imaging with a lateral step size of 3  μm, the intensity, i.e., the amplitude sums of both Fourier transformed camera signals, was processed with custom MATLAB (MathWorks Inc., Natick, United States) software and down sampled to an isotropic voxel size of 5  μm in each spatial direction. Assuming a flat image plane in the 3 mm diameter center of the objective’s 10 mm field of view, this volume was used for determining the shape of the ATR crystal. Due to the axial and lateral resolution of utilized system of approximately 5.5 and 12  μm, respectively, OCT was chosen as a suitable, yet available, optical technique for determining the surface topography of the cone-shape ATR crystal. Hence, it was possible to derive the incident angle of the IR light within the crystal and, thus, the penetration depth within tissue, which is furthermore discussed in the supplements.

### 2.4 4′,6-Diamidino-2-phenylindole dihydrochloride Staining

For fluorescent diamidino-2-phenylindole (DAPI), staining freshly cut tissue surfaces were gently pressed on a glass slide. After a few seconds the tissue was removed and remains on the slide were fixed with paraformaldehyde for 2 min, rinsed twice with dulbecco’s phosphate buffered saline (DPBS) and incubated with DAPI-DPBS solution (1:10.000; DAPI, D8417, Sigma-Aldrich, Saint Louis, United States) for 15 min. After washing the slide with DPBS, fluorescent microscopy with 405-nm laser excitation was performed.

### 2.5 Data Analysis and Classification

Preprocessing prior to analysis and classification of the spectra was performed with the spectroscopy software OPUS (Bruker Optik GmbH, Ettlingen, Germany) routines and involved a linear baseline correction and compensation of atmospheric absorption bands. Further evaluation was performed using the MATLAB Package (Version 7, Math Works Inc. Natick, Massachusetts, United States).

Similar to a previous work, a three-class classification model for normal tissue, tumor tissue, and pancreatitis has been developed.^16^ The classification algorithm is based on the principle of the program “ORS in Georgia.”^19^ The algorithm was slightly modified and used, for example, for the classification of IR spectra of human gliomas.^20^ The procedure, applied on IR spectra, was described in detail elsewhere.^21^ The algorithm first performs an optimized selection of spectral regions for classification. From a previous study, it is known that ∼8 to 10 spectral regions are sufficient to distinguish the three tissue types.^16^ Therefore, the maximum number of spectral regions to be selected by the classification algorithm was set to 12. The width of each spectral region is determined by the algorithm. Supervised classification was performed using the classify function of the MATLAB statistics toolbox followed by leave-one-out cross-validation. The discriminant function of the classify routine was set to “linear.” The classifiers determined by the training set were finally used for the differentiation of spectra of the test set. The training set was built up by a random selection of 25 spectra for each of the 3 classes. The classification algorithm supplied the probability of membership to normal, tumor, or pancreatitis tissue classes both for the training set and the test set. The calculated probability values (between 0 and 1) were transferred to a red-green-blue (RGB) color plot.

### 2.6 Intraoperative ATR IR Fiber Spectroscopy

Resection margins and general samples from freshly resected specimens of three patients were analyzed as described above on a back table within the operating room. Results of spectral analysis were compared to the final pathological report.

### 2.7 Hematoxylin and Eosin (H&E) Staining

After measurements of ATR IR absorption spectra, the examined tissue was frozen. Subsequently, the sample was cryo-sectioned and one representative section (10  μm) was stained by H&E. For this, the defrosted and dried frozen section was treated with 70% ethanol for 30 s. After a short washing step with distilled water, hematoxylin was applied for 3 min, followed by rinsing in distilled water and hydrochloric acid solution. The tissue was blued in warm water for 5 min and subsequently treated with eosin for 30 s. Before embedding the stained section in Entellan™ new (1.07961, Sigma-Aldrich, Saint Louis, United States), rinsing and dehydration with 70% and 90% ethanol, 2-propanol and xylene were performed.

### 2.8 Study Approval

Written informed consent was obtained from all patients prior to surgery, and this study was approved by the Local Ethics Committee (EK 179052018). An additional consent was obtained for the use of photographs in this study.

## 3 Results

### 3.1 Penetration Depth of IR Radiation

**Fig. 1:**
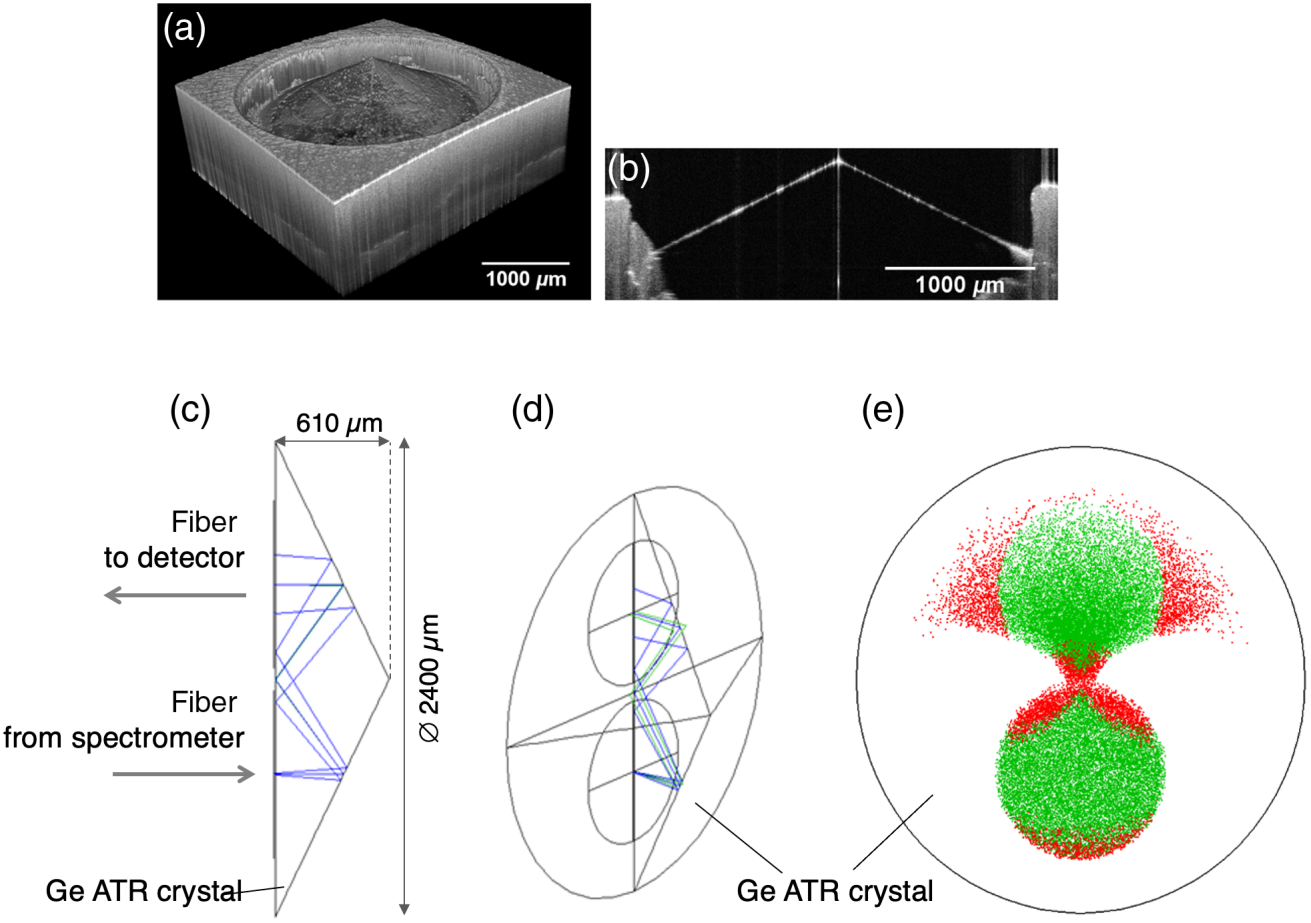
OCT imaging of Ge ATR crystal and ray tracing. (a) OCT image of the Ge ATR crystal tip and (b) a cross-section image of the germanium Ge ATR crystal. (c) Ray tracing indicates a “W” like path. (d) 3D image of ray tracing that was calculated indicating the optical fibers at the base area of the Ge ATR crystal. (e) Calculation of the areas of total reflections on the mantle surface. Rays shown in green can be coupled and guided into the returning fiber to the detector. Rays in red color cannot be guided to the detector.

For the interpretation of the collected spectra, it is important to know the penetration depth of the IR radiation into the tissue since the samples consist of not only multiple cell types (e.g., pancreatic ductal cells, acinar cells, stromal cells, endothelial cells) but the surrounding extracellular matrix (ECM) and blood, as well. In ATR experiments, the light penetrates into the sample, and the spectrum of the sample layer covered by the penetration depth is obtained. Therefore, it is essential to know whether the cells themselves or only EC components are covered by the penetration depth of the light beam. To investigate the precise geometry of the Ge ATR crystal of the fiber probe, we applied OCT to determine the beam paths and to calculate the penetration depth of IR radiation into the tissue sample. Figure 1 shows OCT results [Figs. 1(a) and 1(b)] as well as the beam paths and dimensions of the two areas of total internal reflection.

The optical path depicted in Figs. 1(c) and 1(d) shows that there are three total reflections in the crystal and only the two total reflections on the surface of the cone are used for the spectroscopic measurements. Figure 1(e) shows the size of the areas of total reflections on the mantle surface. The diameter of the surfaces of the total reflection is about 900  μm. The angles of incidence of the total reflection are between 23 deg and 31 deg. The average angle of incidence is 27 deg. This results in penetration depths for the evanescent field is between 0.5 and 1.9  μm. The detailed calculation of the penetration depths is given in Fig. S1 in the Supplementary Material.

**Fig. 2:**
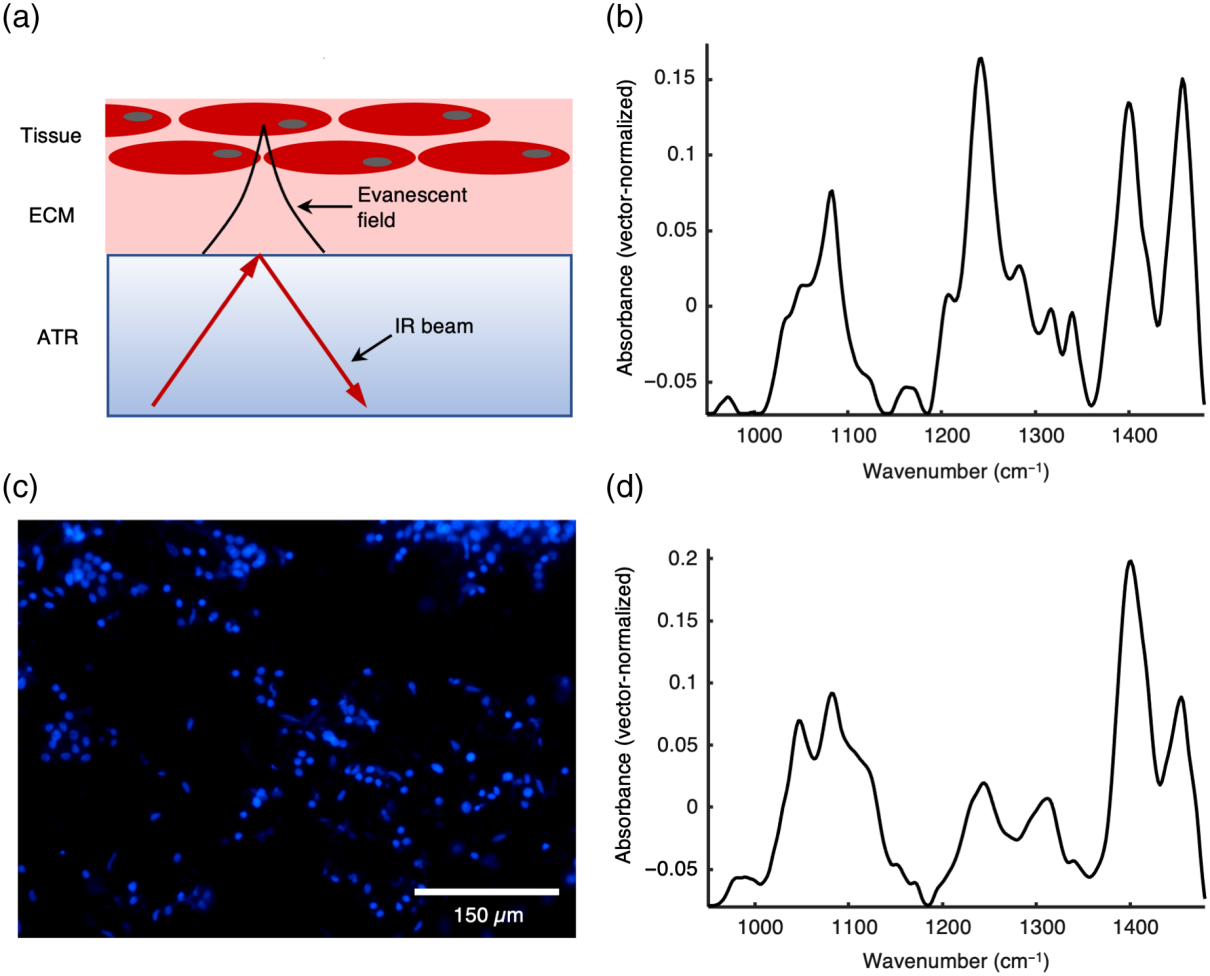
ATR IR absorption spectra of native tissue and tissue smears. (a) Schematic illustration of the ATR IR measurements of tissue. (b) ATR IR absorption spectrum of random native PDAC tissue. (c) Microscopic image of DAPI-stained material left on the slide after removing the tissue. Blue color indicates the nuclei of the cells. (d) ATR IR absorption spectrum of the material left on the ATR crystal after removing the tissue. The demonstrated spectra were baseline-corrected and vector-normalized in a spectral range from 950 to $1480\;{\mathrm{cm}}^{-1}$ to represent the differences not compromised by water absorption bands.

To determine the features being observed from this thickness of sample layers, we compared spectra of native tissue and of the material being left on the Ge ATR crystal after removing the sample. While placing the tissue onto the Ge ATR crystal during the measurements, the ECM fills the space between tissue cells and the Ge ATR crystal [Fig. 2(a)]. However, the thickness of the ECM layer and the components observed in the spectra are crucial for their interpretation.

The IR absorption spectrum of a PDAC tissue was measured [Fig. 2(b)]. Prominent spectral bands located at 1241, 1282, 1316, and $1339\;{\mathrm{cm}}^{-1}$. This spectral pattern can be assigned mainly to plasma proteins including collagen.^22^ In particular, the band at $1241\;{\mathrm{cm}}^{-1}$ can also be assigned to $\nu_{as}({{\mathrm{PO}}_{2}}^{-})$ and thus phosphate groups. However, the $\nu_{s}({{\mathrm{PO}}_{2}}^{-})$ at about $1080\;{\mathrm{cm}}^{-1}$ does not change to the same extent so that nucleic acids as origin of this band can be excluded. It is known that the ECM consists mostly of protein fibers, such as collagen and fibronectin but also of glycosaminoglycans and proteoglycans. The presence of collagen bands in the spectrum indicates that the ECM of the tissue is represented. In a next step, a glass slide was stained with DAPI after placing and removing a PDAC tissue sample [Fig. 2(c)].

Comparing the spectra of the left material after removing the tissue from the ATR crystal and native tissue [Figs. 2(b), 2(d)], it is noticeable that differences between the spectra are visible but in both cases the same spectral bands located at $1047\;{\mathrm{cm}}^{-1}$ assigned to C─O vibrational modes of carbohydrates, $1082\;{\mathrm{cm}}^{-1}$ assigned to ${{\mathrm{PO}}_{2}}^{-}$ vibrational modes of nucleic acids, $1339\;{\mathrm{cm}}^{-1}$ assigned to vibrational modes of C─O and ${\mathrm{CH}}_{2}$ and $1456\;{\mathrm{cm}}^{-1}$ assigned to ${\mathrm{CH}}_{3}$ vibrations being related to proteins are observed.^22^ These findings indicate that the spectra of tissue obtained using the current fiber-based ATR IR method contains the biochemical information of both components, the ECM and cells. It is also supported by the earlier work of Ollesch et al.^11^ that spectral information can be obtained from the tissue using both diamond and Ge ATR crystal. It should be noted that the strong ν(C═O) band at $1744\;{\mathrm{cm}}^{-1}$ of the original raw spectra can also be used for initial detection of predominantly EC medium and the outliers detected here are predominantly from cell-free regions.

### 3.2 Spectral Analysis of Pancreatic Tissue

**Fig. 3:**
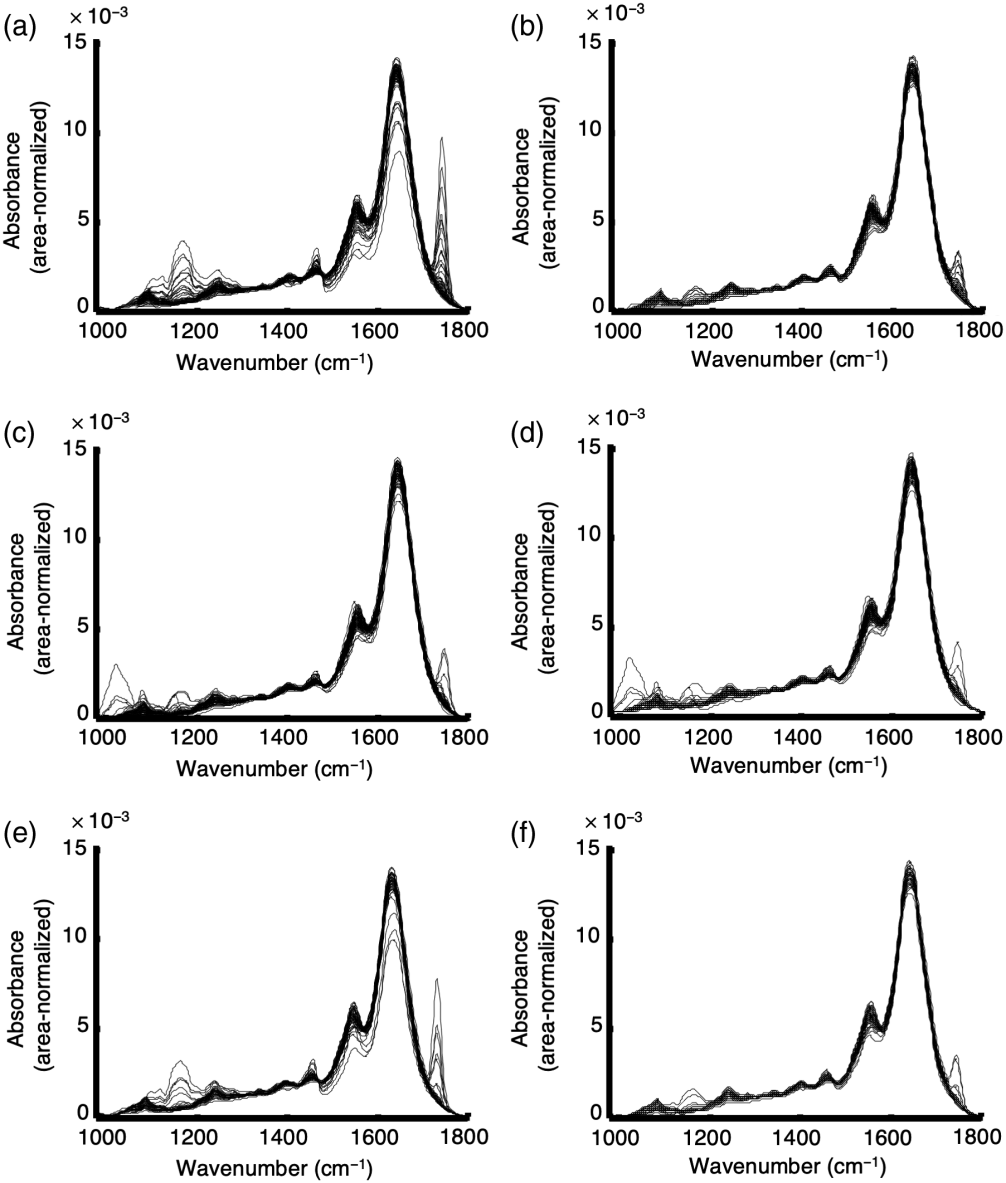
Comparative presentation of total and selected preprocessed spectra. (a) Preprocessed spectra of normal tissue (n=140). (b) Selected preprocessed spectra of normal tissue (n=120). (c) Preprocessed spectra of tumor tissue (n=132). (d) Selected preprocessed spectra of tumor tissue (n=132). (e) Preprocessed spectra of pancreatitis tissue (n=53). (f) Selected preprocessed spectra of pancreatitis tissue (n=50).

Preprocessed and vector-normalized spectra were analyzed regarding spectral profiles that are not associated with pancreatic tissue. These spectra were marked as outliers and removed from the dataset. Furthermore, a few spectra showed strong bands of spectral signatures of lipid-rich tissue suspected to be surrounding tissue entities. Therefore, spectra that showed an absorbance of the band at $1744\;{\mathrm{cm}}^{-1}$ ($A_{1744}$) larger than 0.01 or an absorbance of the band at $1162\;{\mathrm{cm}}^{-1}$ ($A_{1162}$) larger than 0.012 were identified as dominated by fatty acids and subsequently removed from the dataset (Fig. S2 in the Supplementary Material). A two-point baseline was calculated in the spectral ranges from 1120 to $1220\;{\mathrm{cm}}^{-1}$ and from 1710 to $1770\;{\mathrm{cm}}^{-1}$. In total, only 20 spectra of the normal tissue dataset and 3 spectra of the pancreatitis dataset were identified as outliers and were not considered for further analysis. An overview of the number of patient samples and spectra for the preprocessed raw data, selected data and data for the training and test sets for the classification procedure can be found in Table 1. Preprocessed and selected spectra for the three classes of normal, tumor, and pancreatitis tissues were calculated (Fig. 3).

**Table 1:** Summary of the datasets used in this study.

Tissue class	Total data		Selected data		Training set		Test set	
	Samples	Spectra	Samples	Spectra	Samples	Spectra	Samples	Spectra
Normal	41	233	41	213	5	25	37	183
Tumor	40	183	40	183	6	25	34	158
Pancreatitis	11	73	11	70	3	25	8	45

The number of samples corresponds to the number of patients. For some of the patients, both benign and malignant tissue was obtained.

The spectra of all three tissue classes seem to be very similar to each other at first sight. Hence, a successful spectral classification method is dependent on the ability to factor out possible variabilities among the spectra within each class while capturing even slight differences between classes. In particular, supervised classification methods offer an opportunity to identify spectral patterns that are valid within one class even with intraclass variability.

### 3.3 Supervised Classification Model

**Fig. 4:**
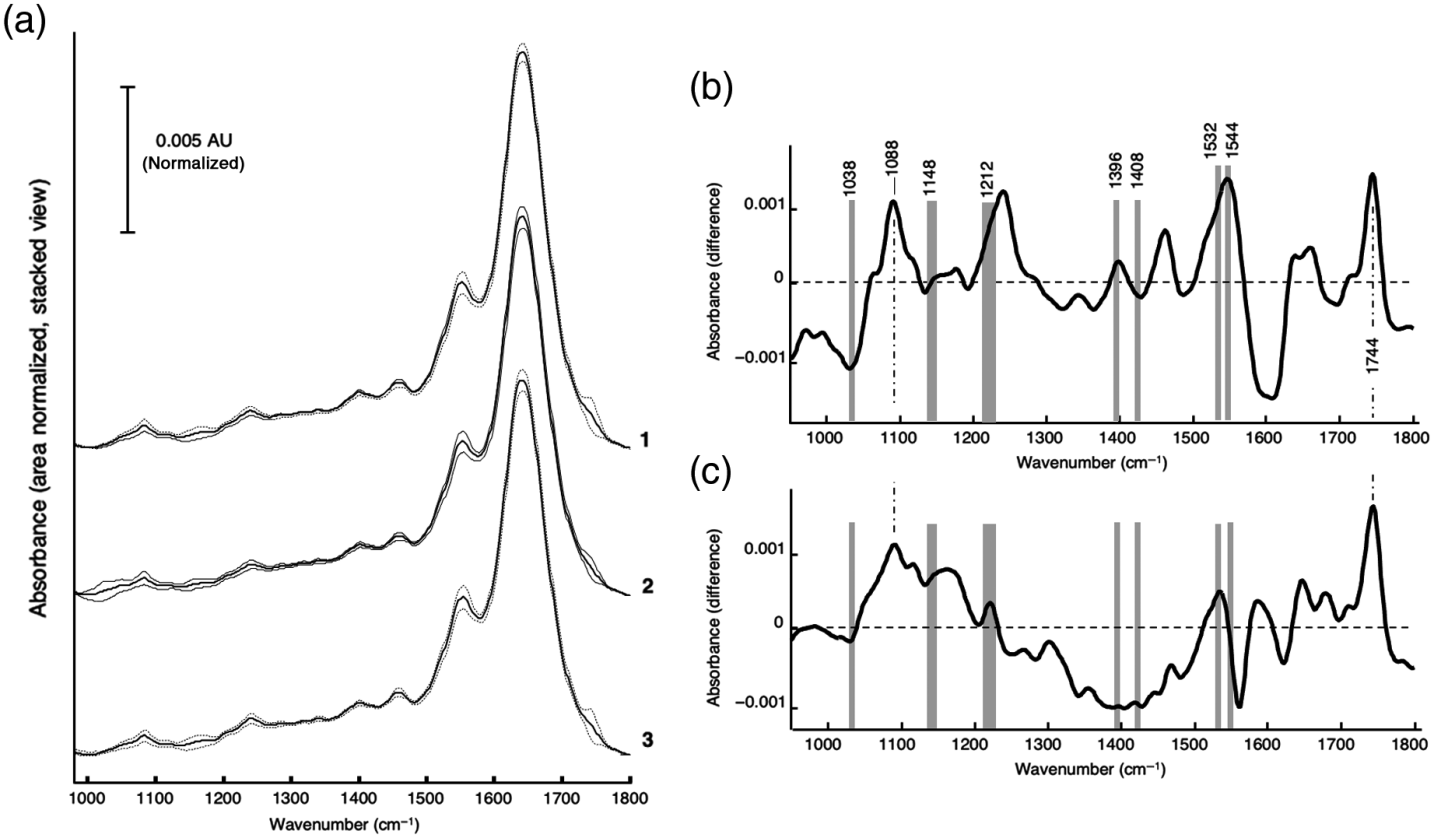
Mean spectra and difference spectra of pancreatic tissue types. Mean spectra together with standard deviation and difference spectra of pancreatic tissue types. (a) Mean spectra (solid line) and standard deviation (dashed lines) of (1) normal, (2) tumor, and (3) pancreatitis tissue. (b) Difference of the mean spectra of “normal” – “tumor”. (c) Difference of the mean spectra of “normal” – “pancreatitis.” Gray bars highlight the selected spectral regions for the classifier that are representative for proper class identification.

For the supervised classification model, 25 spectra of each tissue class were randomly selected for the training set. The mean spectra of “tumor” and “pancreatitis” classes [Fig. 4a] were calculated against those of the “normal” class, and the difference spectra were plotted [Figs. 4(b) and 4(c)]. The classification algorithm chose spectral regions that were representative for the different classes of tissue and these were selected for further distinguishing the different tissue types.

The gray regions depicted in Fig. 4 were identified by the optimal region selection algorithm as the best choice for classification of the spectra into the three classes. The corresponding band assignments for those regions and other general bands are shown in Table S1 in the Supplementary Material.

The difference spectrum of “normal”–“tumor” [Fig. 4(b)] shows stronger signals for normal tissue associated with $\nu_{s}({{\mathrm{PO}}_{2}}^{-})$ modes of nuclei acids at $∼1088\;{\mathrm{cm}}^{-1}$ (not selected for classification), $\nu_{as}({{\mathrm{PO}}_{2}}^{-})$, for a shoulder of the amide III band at $1212\;{\mathrm{cm}}^{-1}$ and for the amide II mode at $1532\;{\mathrm{cm}}^{-1}$. The two selected spectral regions at 1396 and $1408\;{\mathrm{cm}}^{-1}$ suggest variations in the ${\mathrm{CH}}_{x}$ groups of lipids between normal and cancer tissue. Tumor tissue revealed stronger signals at ∼1038 and $1600\;{\mathrm{cm}}^{-1}$ (not selected for classification). An exact assignment of the signals to structural groups is difficult since several vibration modes of different functional groups are superimposed in these spectral ranges. Slightly lower differences of absorbance values were observed in the difference spectrum of “normal”–“pancreatitis” at $∼1212\;{\mathrm{cm}}^{-1}$ [Fig. 4(c)] when comparing with the difference spectrum of “normal”–“tumor.” In addition, the absorbance at $1038\;{\mathrm{cm}}^{-1}$ is stronger in tumor tissue. Considering that vibrational spectroscopy reveals the global biochemical profile of a given tissue section, a precise assignment and interpretation of spectral differences is difficult and a more detailed and molecular analysis for further interpretation is needed. However, it can be concluded that the profiles of proteins and lipids are significantly different among normal, tumor, and pancreatitis tissues. Therefore, the classification approach adopted here offers the immediate prospect of identifying specific diagnostic spectral regions that provide the basis to distinguish among the three tissue types. The spectral regions identified through the region selection algorithm were ascribed to specific tissue components, thus revealing the compositional differences that underlie the spectroscopic-based diagnostic method.

**Fig. 5:**
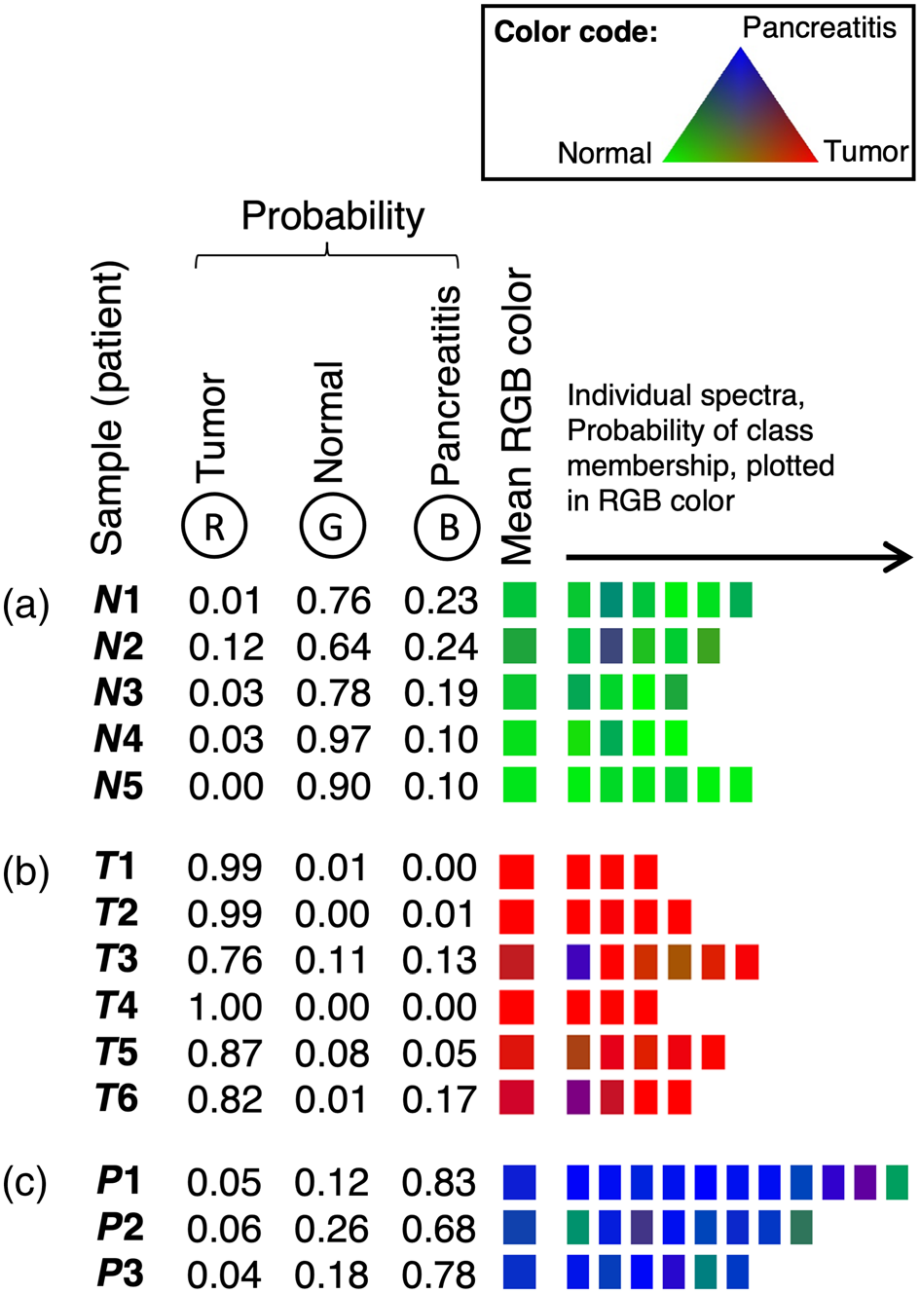
Reclassification of the spectra used for the training set. (a) N1 to N5: samples of normal tissue, (b) T1 to T6: samples of tumor tissue, and (c) P1 to P3: samples of pancreatitis tissue. From each sample, a minimum of three spectra at different areas were recorded. The probabilities of belonging to one of the three classes of “normal”, “tumor,” and “pancreatitis,” calculated by the classifier, are listed and transferred to an RGB color code. The mean RGB color was calculated from the RGB values of the individual spectra.

**Fig. 6:**
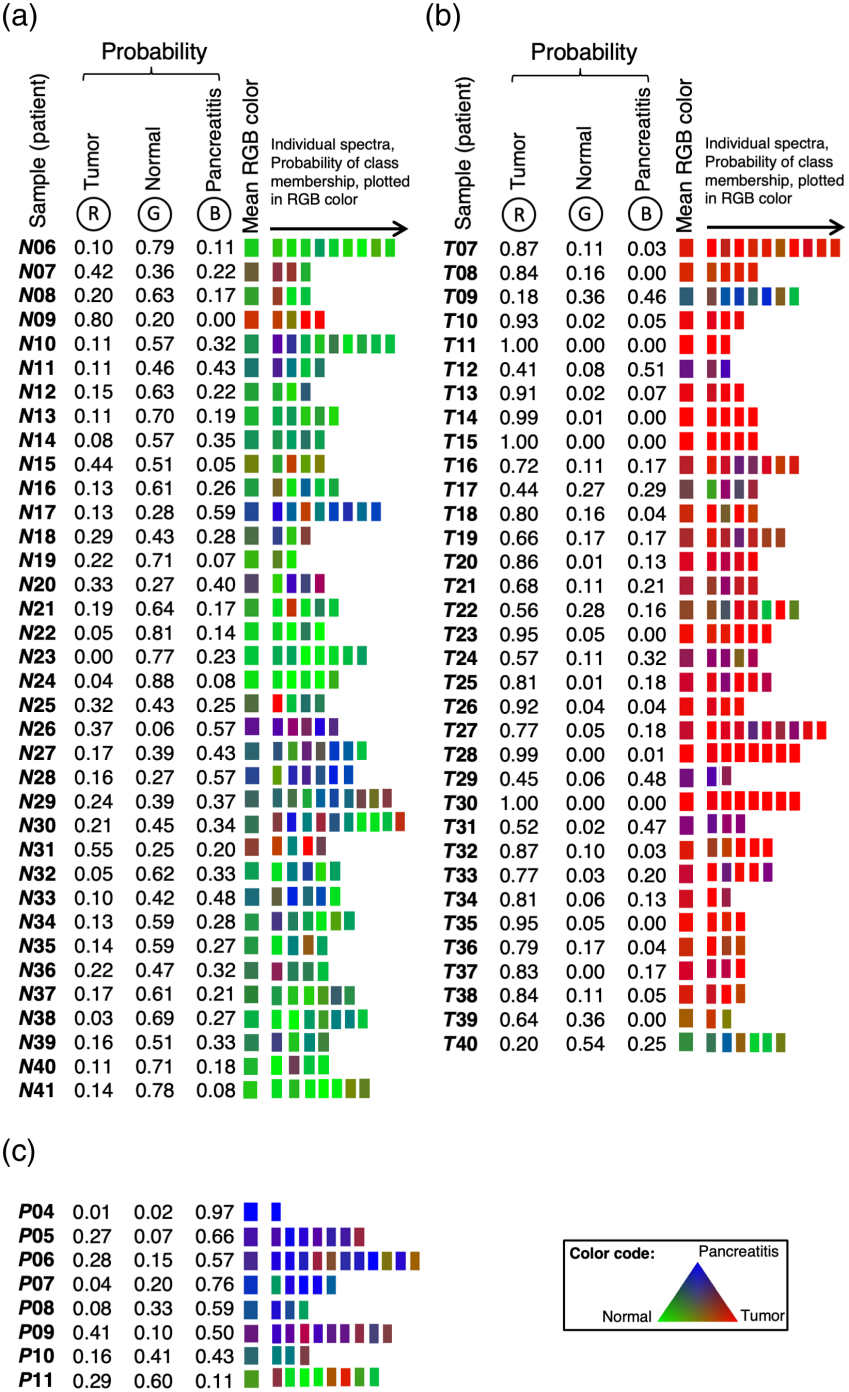
Classification of the test set. (a) N6 to N41: samples of normal tissue, (b) T7 to T40: samples of tumor tissue, and (c) P4 to P11: samples of pancreatitis tissue. From each sample, 1 to 10 spectra at different areas of the specimen were recorded. The probabilities of belonging to a certain tissue class calculated by the classifier are listed and transferred to an RGB color. The mean RGB color was calculated from the RGB values of the individual spectra.

Subsequently, the training set was reclassified using the previously generated classifier and correctly assigned to the specific classes in all samples (Fig. 5). Only a few individual spectra showed a slightly different spectral pattern potentially caused by the macroscopic selection process. However, the class assignment probability score proved the correct classification for the whole sample. Furthermore, using the classifier developed with the training set, the test set samples were assigned (Fig. 6). Most of the individual spectra and samples were classified correctly, indicated by the individual probability for the three classes visually represented by RGB designation. The highest probability defined the class membership. For all classes, only some of the tissue specimens were misclassified. The pathohistological reports were revisited for potential reasons for the algorithm’s misclassification (Table S2 in the Supplementary Material). Possible reasons were misclassifications in the macroscopic selection process, tumor cell infiltration within the resection margin or inflammatory processes within the tumor-surrounding “normal” tissue for example. Further information of all patients is listed in Table S3 in the Supplementary Material.

### 3.4 Intraoperative Case Study

**Fig. 7:**
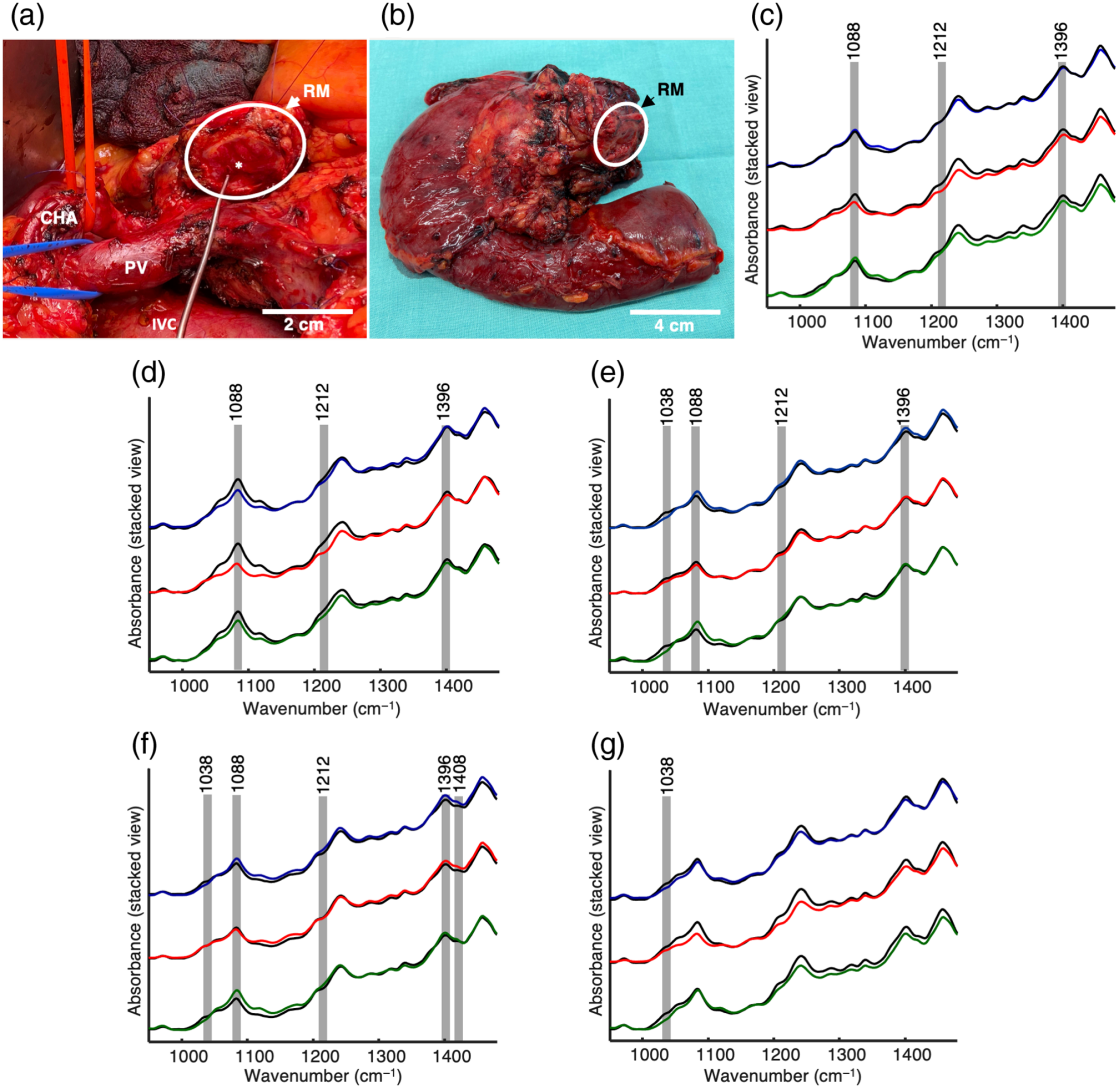
Intraoperative analysis of resected tissues. (a) Intraoperative image of pancreatic head resection *in vivo* (the first case analysis). (b) Resected tissue complex consisting of the pancreatic head and duodenum (the first case analysis). (c) Recorded ATR IR absorption spectrum of the resection margin (black line) compared to mean spectra of pancreatitis (blue line), tumor (red line) and normal pancreatic tissue (green line) (the first case analysis). Recorded ATR IR absorption spectra of (d) resected pancreatitis tissue (black lines) initially considered as normal tissue and (e) resected tumor tissue (black lines) initially considered as pancreatitis tissue compared to mean spectra of pancreatitis (blue line), tumor (red line), and normal pancreatic tissue (green line) (the second case analysis). Visual comparison of the obtained spectra with the mean class spectra reveals the discrepancy between macroscopic assignment and spectroscopic analysis. Recorded ATR IR absorption spectra of (f) resected tumor tissue (black lines) initially considered as normal tissue by surgeon and (g) resected tumor tissue (black lines) initially considered as normal tissue compared to mean spectra of pancreatitis (blue line), tumor (red line), and normal pancreatic tissue (green line) (the third case analysis). Demonstrated spectra were baseline corrected and vector-normalized. Gray bars demonstrate obvious differences between the classes and the analyzed sample following the interclass differences given in Fig. 3. Spectral regions were selected by the classificational algorithm (see Fig. 3), even if the differences seem marginal. RM, resection margin; CHA, common hepatic artery; PV, portal vein; IVC, inferior vena cava; * pancreatic duct.

To establish the potential of an intraoperative application of the fiber-based ATR IR spectroscopy, we performed a case study for three patients receiving a partial pancreatic resection. Measurements were performed as a back table analysis in the operating room. In the first case, a pancreatic head resection was performed on a 70-year-old male patient for an uncertain mass in the pancreas causing cholestasis and unintended weight loss. Resection margins were analyzed with ATR IR fiber spectroscopy on the back table [Figs. 7(a)–7(c)]. The obtained spectra (n=10) were compared with the previously obtained mean spectra of tumor, pancreatitis, and normal pancreatic tissues (Fig. 3). Prospective analysis of the spectra as well as the obtained classifier model assessed the specimen as pancreatitis, which was confirmed by the pathology report as an autoimmune pancreatitis 5 days later.

The second case was a 74-year-old woman receiving a pancreatic head resection due to a lesion in the pancreatic head leading to recurrent vomiting episodes caused by a gastric outlet obstruction. During the surgery two samples of the resected tissue were obtained for the ATR IR spectroscopic analysis. Tissue samples were macroscopically evaluated by the pathologist and assessed as normal and pancreatitis tissue samples. Collected spectra (n=11) of the samples were compared with the mean spectra of tumor, pancreatitis, and normal pancreatic tissue [Figs. 7(d)–7(e)]. The first tissue sample, considered as normal tissue [Fig. 7(d)], was identified as pancreatitis tissue after visual analysis of spectra and application of the developed classification model. The second tissue sample, considered as pancreatitis tissue [Fig. 7(e)], was spectroscopically assigned as tumor tissue. The final pathology report proved that tissue types were correctly identified using fiber-based ATR IR spectroscopy, intraoperatively.

The third patient included in the case study was a 73-year-old man who underwent pancreatic head resection due to a suspected distal cholangiocellular carcinoma infiltrating the pancreas according to computed tomography scans. For intraoperative back table spectroscopic analysis of the resected tissue, two distinct tissue specimens were used. Both were macroscopically evaluated as normal tissue by the surgeon or a pathologist, respectively. After visual analysis of the obtained spectra [Figs. 7(f)–7(g)] as well as applying the previously established classifier model, however, all collected spectra of each sample (n=5 for the first sample; n=4 for the second sample) were assigned to the tumor class. For the first tissue no common pathology report was available since this specimen was not regularly assigned for postoperative histological examinations. For this reason, a frozen section of the measured tissue from this patient was stained by H&E and subsequently evaluated by a pathologist who confirmed malignant cells within the examined tissue (Fig. S3 in the Supplementary Material). The second tissue was identified as pancreatitis tissue with an infiltrating distal cholangiocarcinoma in this setting. Therefore, the tissue classification established by ATR IR spectroscopy was presumably correct.

These results suggest that our proposed method may provide essential assistance for tissue evaluation during surgery and guiding the resection area since the result can be achieved within a minute and reveal relevant tissue information.

## 4 Discussion

This paper shows novel possibilities of the label-free application of ATR IR spectroscopy for delineation of normal pancreatic, timorous, and pancreatitis tissue. Although most applications of IR spectroscopy focus on processed tissue samples, such as frozen, fixed, or dried specimens, we show the application for the unprocessed freshly resected pancreatic tissue. This opens a new opportunity for using the method in a clinical diagnostic setting. The application of a fiber-based probe allows one to examine the tissue under in situ conditions.

While using the described set-up, the penetration depth of the IR radiation into the sample varies from 0.5 to 1.9  μm. In this case, both information of the biochemical composition of the ECM and cellular components of the tissue are observed.

Within the difference spectrum “normal”–“tumor,” the amide III and amide II signals indicate a higher content of proteins in the normal tissue; however, a stronger signal for amide I was not observed in this tissue type. This finding appears to be in contrast to studies that report that pancreatic cancer is associated with significantly increased circulating levels of collagen, particularly type IV collagen.^23^ However, not only classical PDAC but also other tumor entities infiltrating or located in the pancreas were analyzed. In general, the protein and lipid profiles of the tissue spectra are significantly altered within the classes. Based on the characteristic regions in the spectra, a supervised classification model was developed for delineation of pancreatic tissues that allows a valid discrimination among normal pancreatic, timorous, and pancreatitis tissues with established class probability values for each sample. Very few samples of the tumor and normal tissue test set were not classified properly. Potential explanations of the algorithm’s error were given (Table S2 in the Supplementary Material). Indeed, a training set with 25 spectra for each class might be too small to represent the complex heterogeneity of biological samples. Nevertheless, the study underlines the potential of fiber-based ATR IR spectroscopy for a precise application in surgical oncology. From a technical point of view, ATR IR fiber spectroscopy can be easily implemented in the clinical workflow, as the portable device can be placed on any table. The case study provides an opportunity for the path into surgical routine use that is easily manageable and gives stable results. Thus, we envision to spectroscopically analyze pancreatic tissue in situ in future intraoperative applications.

For the coverage of intraclass variabilities, further biochemical analysis and a wider range of training set spectra are needed. In general, the feasibility of this method as an intraoperative back table application opens a broad range of usage for precise oncological resections in pancreatic surgery and leads to an improvement of patient-oriented treatment modalities in solid cancer entities.

## Code, Data, and Materials Availability

The datasets generated and analyzed during the current study are available from the corresponding author upon request.
