# Supplementary material for: Fast and label-free intraoperative discrimination of malignant pancreatic tissue by attenuated total reflection infrared spectroscopy
Source: J Biomed Opt. 2023 Apr 28;28(4):045004. doi: 10.1117/1.JBO.28.4.045004 (PMC10142231; doi:10.1117/1.JBO.28.4.045004)
Supplement: Supplementary file 1 [file JBO_028_045004_SD001.pdf]

Fast and label-free intraoperative discrimination of malignant pancreatic tissue by attenuated total reflection infrared spectroscopy

Rimante Bandzeviciute[1,2], Gerald Steiner[3], Katja Liedel[2,4], Jonas Golde[3], Edmund Koch[3], Thilo Welsch[5], Christoph Kahlert[2,4], Daniel E. Stange[2,4], Marius Distler[2,4], Jürgen Weitz[2,4], Justinas Ceponkus[1], Valdas Sablinskas[1], and Christian Teske[2,4,*]

[1] Institute of Chemical Physics, Faculty of Physics, Vilnius University, Vilnius, Lithuania

[2] Department of Visceral, Thoracic and Vascular Surgery, University Hospital Carl Gustav Carus, Technische Universität Dresden, Dresden, Germany

[3] Department of Anaesthesiology and Critical Care Medicine, Clinical Sensoring and Monitoring, Faculty of Medicine, Technische Universität Dresden, Dresden, Germany

[4] National Center for Tumor Diseases (NCT/UCC), Dresden, Germany; German Cancer Research Center (DKFZ), Heidelberg, Germany; Faculty of Medicine and University Hospital Carl Gustav Carus, Technische Universität Dresden, Dresden, Germany; Helmholtz-Zentrum Dresden-Rossendorf (HZDR), Dresden, Germany

[5] Department of General, Visceral und Thoracic Surgery, St. Elisabethen-Klinikum Ravensburg, Academic Teaching Hospital of the University of Ulm, Ravensburg, Germany

Supplementary material

*** Corresponding author:**

Christian Teske, MD

Department of Visceral, Thoracic and Vascular Surgery

University Hospital Carl Gustav Carus

Technische Universität Dresden

Fetscherstraße 74

01307 Dresden

Germany

Tel: +49 351 458 11909

Email: christian.teske@ukdd.de

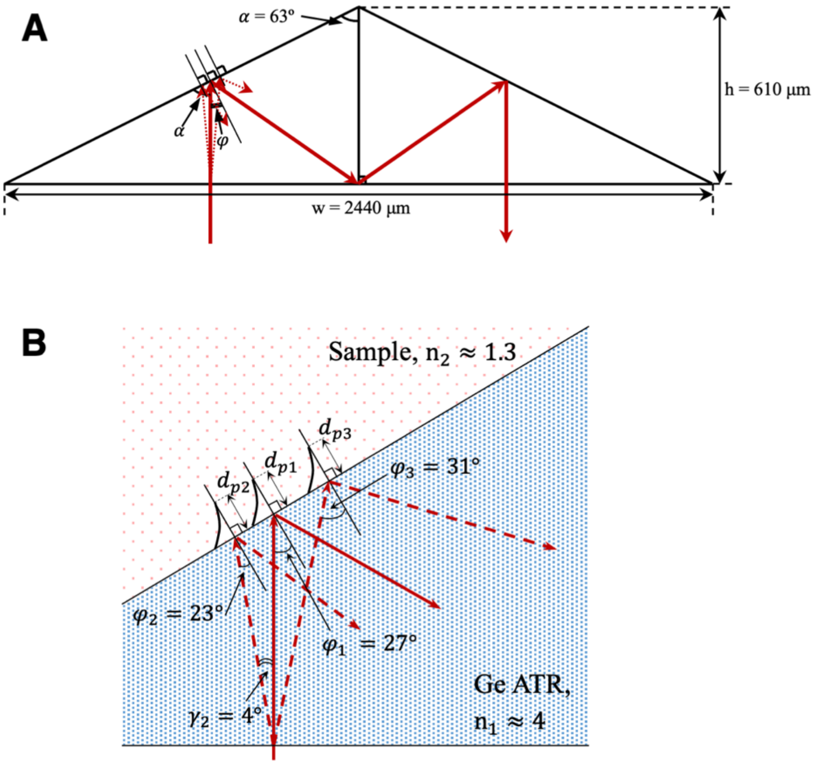

C

Half of the top angle of the crystal cone (α):

$$\mathrm{tg}\alpha = \frac{w/2}{h} = 2, \quad \alpha = 63°$$

The angle of incidence (φ_1) to the surface between the crystal and the sample:

$$\varphi_1 = 90° - \alpha = 27°$$

Depth of penetration of IR beam to the sample:

$$d_p = \frac{1}{2\pi\tilde{\nu}n_1\sqrt{\sin^2\varphi - \left(\frac{n_2}{n_1}\right)^2}}$$

Depth of penetration (d_{p1}) for wavenumbers 950 and 1800 cm^{-1} when the angle of incidence $\varphi_1 = 27°$, $\varphi_2 = 23°$ and $\varphi_3 = 31°$:

	$\tilde{\nu} = 950\ cm^{-1}$	$\tilde{\nu} = 1800\ cm^{-1}$
$\varphi_1 = 27°$	$d_{p1} = 1.3\ \mu m$	$d_{p1} = 0.7\ \mu m$
$\varphi_2 = 23°$	$d_{p2} = 1.9\ \mu m$	$d_{p2} = 1.0\ \mu m$
$\varphi_3 = 31°$	$d_{p3} = 1.0\ \mu m$	$d_{p3} = 0.5\ \mu m$

Supplementary Figure 1 Beam path and penetration depth calculations.

(**A**) The beam path inside the ATR crystal was calculated and is presented accordingly. Solid red lines indicate the perpendicular beam path from the fiber to the ATR crystal surface. Dashed red lines indicate the beam path due to the numerical aperture of the fiber (the effective numerical aperture of the fiber is 0.3). α half of the top angle of the prism cone, φ angle of incidence to the surface between the crystal and the sample, h height, w width of the crystal. **(B)** Depth of penetration for different angles of incidence φ. Angle of incidence φ_1=27˚ when the beam path from the fiber to the ATR crystal surface is perpendicular, angle of incidence φ_2=23˚ and φ_3=31˚ when the beam due to numerical aperture of the fiber bends. d_{p1}, d_{p2} and d_{p3} depth of penetration respectively for angles of incidence φ_1, φ_2 and φ_3. n_1, n_2 refractive indexes for germanium (Ge) and blood. **(C)** Calculation of depth of penetration for different wavenumbers and angles of incidence. $\tilde{\nu}$ wavenumber.

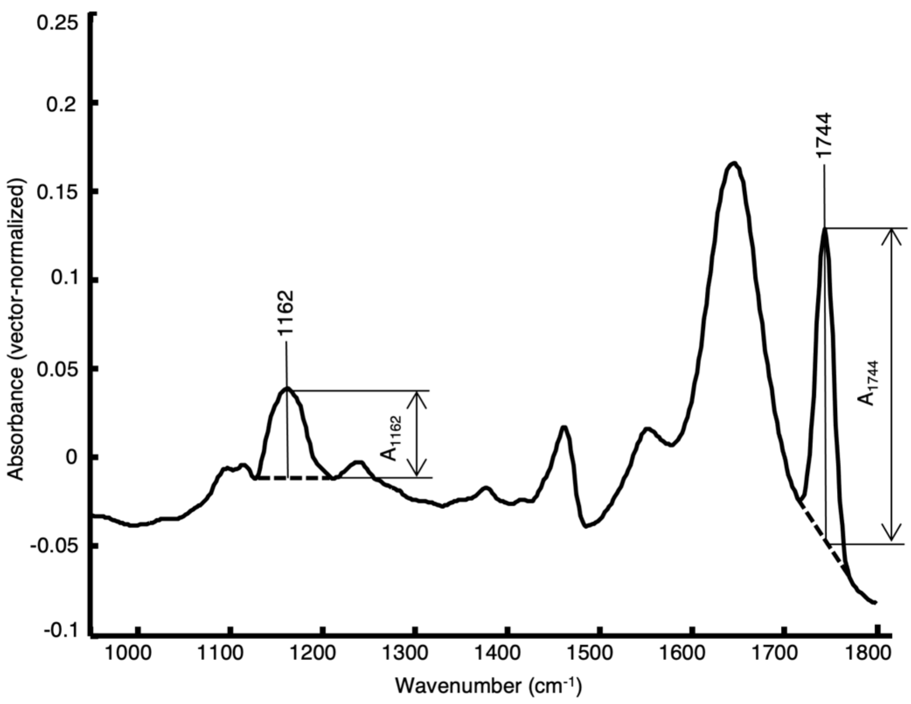

Supplementary Figure 2 Illustration of exclusion criteria for spectra.

Calculated absorbances at 1162 cm^{-1} and 1744 cm^{-1} identifying spectra associated with high amounts of fatty tissue are indicated. Respective spectra were excluded from further analysis.

Spectral position (cm^{-1})	Assignment
1744	ν(C=O)
~1640	Amide I
~ 1600	ν(C-N), δ(C-N), δ(N-H)
~ 1550	Amide II
1532	ν(C=N) ν(C=C)
1458	δ(CH_3)
1417	ν(C-N), δ(C-N), δ(N-H)
1396	δ(CH_3), δ(CH_2)
1408	δ(CH_3)
1339	ν(C-O), δ(CH_2)
1240	Amide III, ν(C-N), ν(PO_2^-)
1212	ν(PO_2^-)
1206	Amide III, ν(PO_2^-),
1173	ν(C-O)
1148	ν(C-O) of carbohydrates, δ(CH_3)
1088	ν_s(PO_2^-) of DNA and RNA
1082	ν_s(PO_2^-) of DNA and RNA
1047	ν(C-O) of carbohydrates, glycogen
1038	ν(C-C) skeletal, ν(CH_2OH), ν(C-O), δ(C-O)
1019	ν(C-O), δ(C-O)
971	ν(PO_2^-)

Supplementary Table 1 Assignment of IR absorption spectral bands.

Spectral IR absorption bands of pancreatic tissue in the spectral region from 950 cm^{-1} to 1800 cm^{-1} and their molecular assignments[16]. δ deformation, ν stretching, s symmetric.

# Sample	Comments in the histological report
N07	Patient received adjuvant chemotherapy (CTx) for a previous gastric cancer several years before and neoadjuvant CTx for the recurrent disease. Cancer cells spread separately into pancreatic tissue – macroscopic "normal" tissue might be invaded by singular gastric cancer cells.
N09	Patient received neoadjuvant CTx, tumor was accompanied by lymphangiosis carcinomatosa, and tumor bulks reached the resection margin – macroscopic "normal" tissue might be coupled with microscopic tumor bulks.
N17	Patient had chronic pancreatitis with inflammatory tissue reaching into the resection margin – macroscopic "normal" tissue might be associated with histologically inflammatory tissue.
N20	Patient had a rare acinar cell carcinoma of the pancreatic tail infiltrating into the spleen, stomach and vascular structures. The underlying pathology might be peri-tumoral pancreatitis.
N26	The analyzed specimen was H&E-stained and re-evaluated with inflammatory tissue within the section typical for pancreatitis.
N27	The analyzed specimen was H&E-stained and re-evaluated with inflammatory tissue within the section typical for pancreatitis.
N31	Patient had an undifferentiated carcinoma with diffuse tumor cell infiltration within the resection margin. Macroscopic "normal" tissue might be coupled with microscopic tumor bulks.
N33	Patient received neoadjuvant chemotherapy before surgical resection. Pathological report states chronic pancreatitis within the tumor-surrounding tissue.
T09	Patient had cholangiocellular carcinoma with microscopic intraepithelial neoplasms reaching into the resection margin – it is macroscopically difficult to differentiate between normal and tumor tissues, and the pathologic report did not mention pancreatitis.
T12	The tissue sample was acquired by punch biopsy during index surgery, and consecutive punch biopsies were also microscopically classified as benign. However, pathologically proven PDAC was resected during subsequent operation.
T29	Patient received neoadjuvant chemotherapy before surgical resection. Most of the tumor cells were necrotic and avital. Peritumoral pancreatic tissue displayed fibrosis.
T40	The analyzed specimen was H&E-stained and re-evaluated with no tumor cells within the section.

Supplementary Table 2 Retrospective review of the misclassified samples.

Pathological reports of misclassified normal (N) and tumor (T) tissue samples of the test set were reviewed for potential explanation of the algorithm error.

Original patient number	Training set - Figure label	Test set - Figure label	Sex	Age	Pre-treatment	Diagnosis
Patient 1		N5, T7	Male	52	No	Pancreatic neuroendocrine tumour
Patient 2	P1	P4	Male	49	No	Chronic pancreatitis
Patient 3	T1	N7	Female	65	Yes	Recurrent stomach cancer in pancreatic head
Patient 4	N1	T8	Male	75	No	Metastasis of renal cell carcinoma in pancreatic head
Patient 5		N8, T9	Male	84	No	Distal cholangiocelluar carcinoma
Patient 6		N9, T10	Female	59	Yes	PDAC
Patient 7	N2		Female	79	No	PDAC
Patient 8		N10, T11	Male	82	No	PDAC
Patient 9		T12	Male	61	No	Pancreatic biopsy, pathology report without malignancy
Patient 10	P2	N11	Male	77	No	Chronic pancreatitis
Patient 11		N12	Female	71	No	Normal pancreas
Patient 12	T2		Male	74	No	PDAC
Patient 13		N13, T13	Male	62	No	PDAC
Patient 14		P4	Male	67	No	Chronic pancreatitis
Patient 15		T14	Male	77	Yes	PDAC
Patient 16	N3	T15	Male	67	Yes	PDAC
Patient 17		N14, T16	Male	59	No	Acinar cell carcinoma
Patient 18		P5	Male	34	No	Chronic pancreatitis
Patient 19	T3		Male	63	No	PDAC
Patient 20		N15, T17	Female	68	Yes	PDAC
Patient 21		N16, T18	Male	64	No	PDAC
Patient 22		N17, T19	Male	62	Yes	PDAC
Patient 23		N18, T20	Female	57	No	PDAC
Patient 24	N4, T4		Male	55	Yes	PDAC
Patient 25		N19, T21	Male	69	No	Distal cholangiocelluar carcinoma
Patient 26		P6	Male	60	No	Chronic pancreatitis
Patient 27		N20, P7	Male	68	No	Chronic pancreatitis
Patient 28	P3		Male	52	No	Chronic pancreatitis
Patient 29		T21, T22	Male	64	Yes	PDAC
Patient 30	T5	N21	Female	19	No	Solid-pseudopapillary neoplasm of the pancreas
Patient 31		N22, T23	Male	70	No	Pancreatic neuroendocrine tumour
Patient 32		N23	Male	63	Yes	PDAC
Patient 33	N5, T6		Male	76	No	Intraductal papillary mucinous neoplasm (IPMN, precursor lesion)
Patient 34		T24	Male	60	No	Pancreatic colloidal carcinoma
Patient 35		N24, T25	Female	61	No	Serous cystic adenoma (benign lesion)
Patient 36		T26	Male	69	No	Pancreatic neuroendocrine tumour
Patient 37		N25, T27	Female	79	No	PDAC
Patient 38		P09	Male	65	No	Chronic pancreatitis
Patient 39		N26, T28	Female	73	No	Intraductal papillary mucinous neoplasm (IPMN, precursor lesion)

Patient 40		N27, T29	Male	75	Yes	PDAC
Patient 41		N28	Female	63	No	Gall bladder carcinoma
Patient 42		N29, T30	Male	73	No	Pancreatic colloidal carcinoma
Patient 43		N30, T31	Female	78	No	PDAC
Patient 44		case study (1)	Male	70	No	Autoimmune pancreatitis
Patient 49		N31, T32	Female	51	No	Undifferentiated carcinoma
Patient 50		N32, T33	Male	70	No	PDAC
Patient 51		N33, T34	Male	67	Yes	PDAC
Patient 52		N34	Male	66	No	Neuroendocrine tumor of the pancreas
Patient 53		P10	Male	71	No	PDAC
Patient 55		N35, T35	Male	79	No	PDAC
Patient 56		N36, T36	Male	72	No	Ampullary carcinoma
Patient 58		N37, T37	Female	72	No	PDAC
Patient 59		N38, P11	Male	51	No	PDAC
Patient 60		case study (2)	Female	74	No	PDAC
Patient 61		case study (3)	Male	73	Yes (for previous B cell lymphoma	Distal cholangiocelluar carcinoma
Patient 62		N39, T38	Male	55	No	PDAC
Patient 63		N40, T39	Male	80	No	PDAC (adenosquamous carcinoma)
Patient 64		N41, T40	Male	82	Yes	PDAC

Supplementary Table 3 Patient information of the cohort under study.

Detailed patients’ information included in the study.

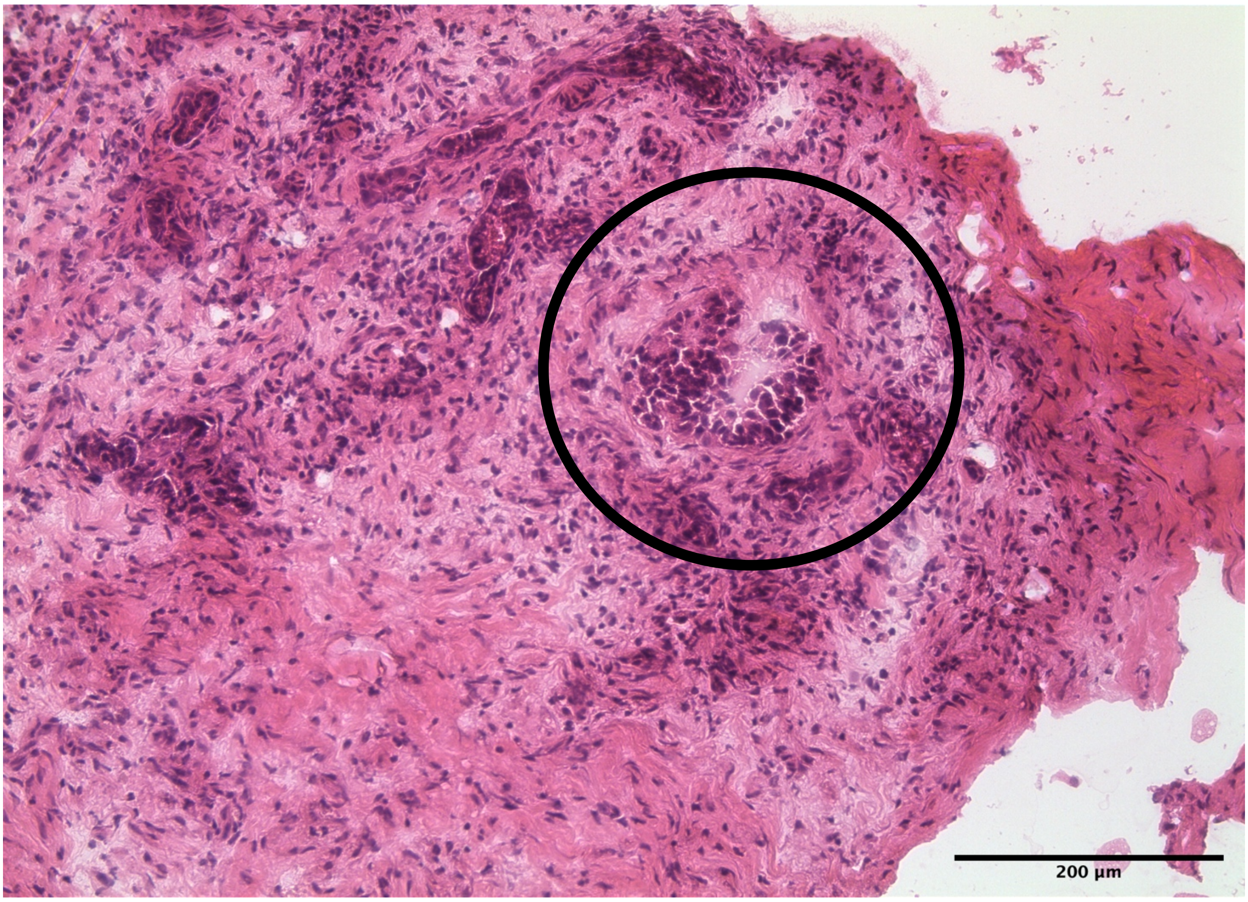

Supplementary Figure 3 Detection of malignant structures within sampled tissue of patient 40.

A frozen section of the first spectroscopically analyzed tissue of patient 40 was treated H&E-stained and subsequently evaluated by a pathologist. The circled area marks a representative malignant lesion identified by the pathologist.
